# Substoichiometry in the Ba-Cu and Ba-Cu-O phase diagrams

**DOI:** 10.1063/4.0000805

**Published:** 2025-10-27

**Authors:** Avinash Kiran, Justin L Andrews

**Affiliations:** Purdue university, West Lafayette, Indiana, USA

## Abstract

Electrides, which are a unique class of materials featuring anionic electrons (*i.e.*, *Farbe* centers) localized at discrete crystallographic sites, provide an ideal platform for systematically investigating electron correlation. These compounds, often identified by unconventional stoichiometries such as [Ti_2_O]^²+^[(e^−^)_2_]^²−^, [Hf_2_S]^²+^[(e^−^)_2_]^²−^, [Ca_2_N]^+^(e^−^)^−^, and [Ca_12_Al_14_O_32_]^²+^[(e^−^)_2_]^²−^, exhibit intriguing electronic properties that remain largely unexplored. Here, we present our recent efforts to investigate the electrical behavior of cupride (Cu^−^) compounds within the Ba-Cu binary phase space. Our work has led to the discovery of several previously unknown phases and structure types, including: (1) Ba_20_Cu_14_O_5_, an pseudo anti-Ruddlesden-Popper phase where anionic Cu^−^ is coordinated by Ba^²+^ in a square planar configuration; (2) a novel ∼Ba_7_O_3_ electride phase, featuring Ba_18_O_5_ clusters with an anti-Lindqvist (W_5_O_18_) geometry; and (3) BaCu_5_, a previously unidentified intermetallic phase that is isoelectronic to SrCu_5_ and CaCu_5_ but distinguished by infinite Ba-Ba zigzag chains. These findings expand our understanding of low-dimensional excess electron compounds and their potential applications in electronic and quantum materials.

